# P-415. Monitoring Antibiotic Use: A Study on Prescriptive Appropriateness in a Pediatric Infectious Disease Unit

**DOI:** 10.1093/ofid/ofaf695.632

**Published:** 2026-01-11

**Authors:** Maria Sole Valentino, Marta Stracuzzi, Chiara Bassi, Roberta Caiazzo, Crescenzo Coppola, Daniela David, Raffaella Di Tonno, Francesca Marinacci, Anna Markowich, Francesca Musto, Marc Garcia-Lorenzo, Vania GIacomet

**Affiliations:** Pediatric Infectious Disease Unit, Luigi Sacco Hospital, University of Milan, Milan, Italy, Milan, Lombardia, Italy; Pediatric infectious diseases unit, ASST FBF Sacco – University of Milan, Milano, Lombardia, Italy; University of Milan, Milano, Lombardia, Italy; Pediatric infectious diseases unit, ASST FBF Sacco – University of Milan, Milano, Lombardia, Italy; Pediatric infectious diseases unit, ASST FBF Sacco – University of Milan, Milano, Lombardia, Italy; Pediatric infectious diseases unit, ASST FBF Sacco – University of Milan, Milano, Lombardia, Italy; Pediatric infectious diseases unit, ASST FBF Sacco – University of Milan, Milano, Lombardia, Italy; Pediatric infectious diseases unit, ASST FBF Sacco – University of Milan, Milano, Lombardia, Italy; Pediatric infectious diseases unit, ASST FBF Sacco – University of Milan, Milano, Lombardia, Italy; Pediatric infectious diseases unit, ASST FBF Sacco – University of Milan, Milano, Lombardia, Italy; Pediatric infectious diseases unit, ASST FBF Sacco – University of Milan, Milano, Lombardia, Italy; Pediatric infectious diseases unit, ASST FBF Sacco – University of Milan, Milano, Lombardia, Italy

## Abstract

**Background:**

Antibiotic resistance is a growing phenomenon, partly due to the inappropriate use of these drugs. Although antibiotics are among the most prescribed medications in pediatrics, data on prescribing appropriateness in the hospital setting for this population remain limited. The most recent AIFA (Italian Medicines Agency) report on antibiotic use in Italy (2023) provides general data on hospital prescriptions, without stratification by department type.

**Figure:**
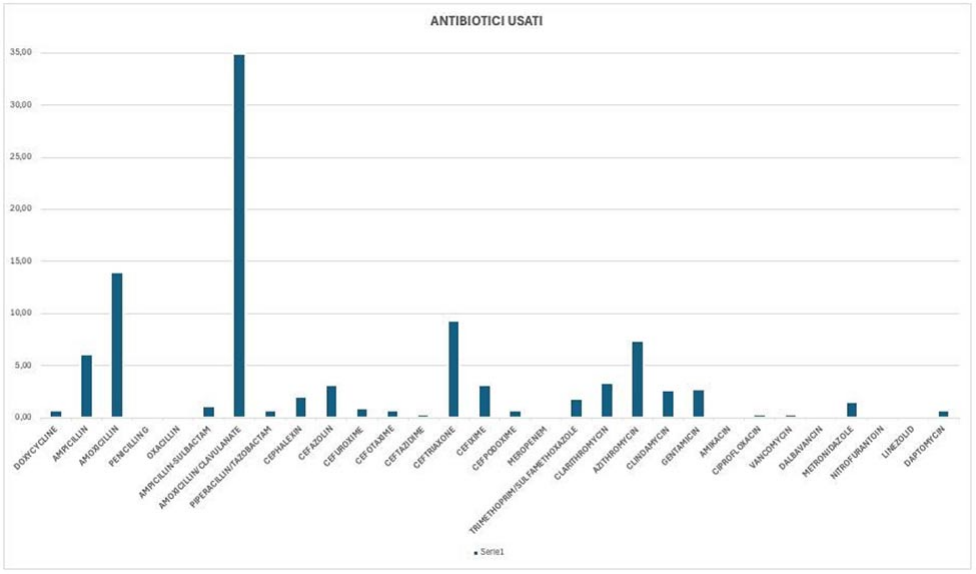

**Figure:**
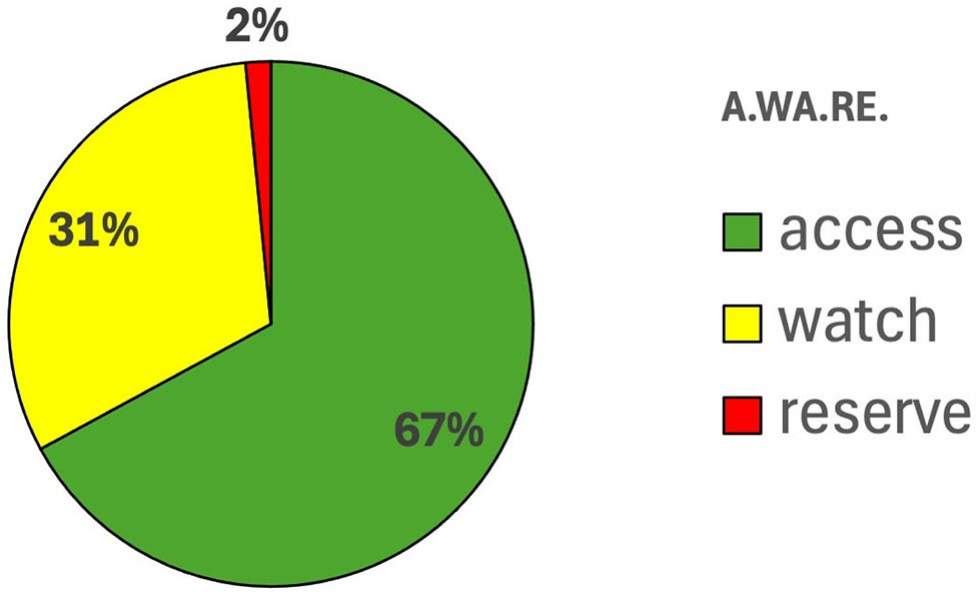

**Methods:**

We conducted a retrospective observational study analyzing the appropriateness of antibiotic therapy prescriptions in our Pediatric Infectious Disease Unit at Luigi Sacco Hospital. Our team implements an antimicrobial stewardship program focused on audits, consultations, and guideline use, including the WHO-promoted A.Wa.Re manual. We included all patients admitted to our department between March 2023 and November 2024 who received antibiotic therapy, evaluating the prescribed molecule, dosage, and treatment duration against national and international guidelines.

**Figure:**
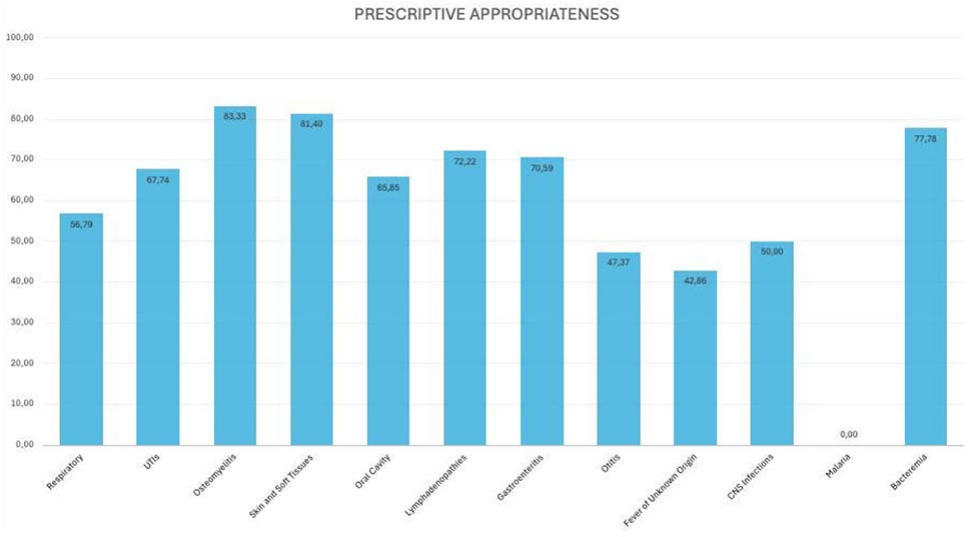

**Results:**

Out of 705 hospitalized patients, 235 received at least one antibiotic. The highest prescription rate was for respiratory diseases, and the most commonly prescribed antibiotic was amoxicillin-clavulanate (Fig.1). Overall, the prescribed antibiotic was appropriate for the condition in 75% of cases, treatment duration was appropriate in 75%, and dosage in 97%. According to the A.Wa.Re classification, 67% of antibiotics prescribed fell under the Access group, 31% under Watch, and 2% under Reserve—aligned with WHO targets, which recommend at least 60% Access antibiotic use in the general population (Fig.2). When analyzing by pathology, guideline adherence was highest for osteomyelitis (83%) and lowest for malaria (0%) (Fig.3). Regarding administration route, 71% of patients received at least one intravenous antibiotic therapy; the average IV therapy duration was 5 days.

**Conclusion:**

Active surveillance of antibiotic therapy is essential for optimizing prescription appropriateness. In this context, the A.Wa.Re classification offers an easily applicable guide for treatment planning. Furthermore, data analysis can be a valuable tool to identify critical points in therapeutic management and implement improvement strategies.

**Disclosures:**

All Authors: No reported disclosures

